# Trastuzumab-induced corneal ulceration: successful no-drug treatment of a “blind” side effect in a case report

**DOI:** 10.1186/s12885-015-1969-3

**Published:** 2015-12-16

**Authors:** A. Orlandi, R. Fasciani, A. Cassano, A. Agresta, M. A. Calegari, A. Caporossi, C. Barone

**Affiliations:** 1 Division of Medical Oncology, Department of Internal Medicine, Catholic University of Sacred Heart, Rome, Italy; 2 Institute of Ophthalmology, Catholic University of Sacred Heart, Rome, Italy

**Keywords:** Trastuzumab, HER2, Limbal lesion, Corneal erosion

## Abstract

**Background:**

We report the first successful treatment of limbal lesions and corneal erosion experienced by a breast cancer patient undergoing trastuzumab treatment.

**Case presentation:**

A 49-year-old Caucasian woman with early stage breast cancer was treated with adjuvant trastuzumab and subsequently showed persistent bilateral corneal marginal infiltrates resistant to topical steroid and antibiotic treatment. Autologous serum was applied in the conjunctival sac as an experimental treatment to antagonize the inhibitory effect of the HER2 receptor antibody on the corneal epithelial cells. Topical application of autologous serum led to rapid improvement of the ulcerative keratitis, with complete healing of the corneal defect after 7 days. Continued administration of the serum allowed the resumption of trastuzumab therapy without any further side effects.

**Conclusions:**

Persistent bilateral corneal marginal infiltrates may occasionally arise as a side effect of trastuzumab treatment. Topical medication with autologous serum may be an effective therapeutic option for the ocular side effects of trastuzumab therapy.

## Background

Trastuzumab, a monoclonal antibody that targets breast cancers which overexpress the HER2 receptor, improves the response rate, time to progression, and overall survival of both metastatic and adjuvant patients [1–4]. Recently, trastuzumab was also shown to be effective in the treatment of metastatic HER2+ gastric cancer [5]. Clinically, the most important side effect of trastuzumab is cardiotoxicity, which is reported in 2.6 to 4.5 % of patients [6]. This side effect remains the only limiting factor in the use of trastuzumab and may appear as an asymptomatic decrease in the left ventricular ejection fraction or symptomatic congestive heart failure [7, 8]. The pathophysiology, which differs from the cardiotoxicity of anthracyclines, is still poorly understood but the important physiological role played by the HER2 pathway in the function of cardiomyocytes is well established [9].

The HER family of receptor tyrosine kinases (EGFR, also termed ErbB1/HER1, ErbB2/Neu/HER2, ErbB3/HER3, and ErbB4/HER4) and their ligands, have been shown to be involved in cell differentiation, proliferation, migration, and carcinogenesis [10, 11]. HER1, HER2 and HER3 are also expressed by corneal, limbal and conjunctival epithelia [12]. Ocular side effects of trastuzumab in human subjects are not commonly observed [13]. In the GeparQuattro study, conjunctivitis was reported as a side effect in 2.5 % of patients [14]. In a different study, using a related drug, trastuzumab-DM1 which is a HER2 antibody-cytotoxic conjugate, ocular side effects were reported for 28 % of patients [15]. These side effects, which were generally mild and tolerable, included: dry eyes, increased lacrimation, mild subjective blurring of vision, and conjunctivitis. ErbB2 serves as a critical component that couples the ErbB receptor to the migration machinery of corneal epithelial cells [16]. Here we report the successful treatment of progressive corneal ulcerative damage which arose due to treatment with trastuzumab.

## Case presentation

A 49 year old Caucasian woman with a ductal carcinoma of the right breast (pT2N1, ER+, PgR+, HER2+ IIC, Ki 67 70 %), after external quadrantectomy, received sequential adjuvant treatment of adriamycin and cyclophosphamide for four cycles, as well as four cycles of docetaxel and trastuzumab, and radiation on the residual mammary gland. This was then followed by 14 additional treatments with trastuzumab every 3 weeks as monotherapy. The patient did not suffer any co-morbidities and was not taking any other medications during treatment. The cytotoxic treatment was well tolerated; however, 10 days after the 12th dose of trastuzumab in monotherapy the patient complained of burning and watery eyes, a sensation of a foreign body in both eyes, although mainly in the right eye, and impaired vision. These sensations were not due to mechanical trauma to the eyes.

**Fig. 1:**
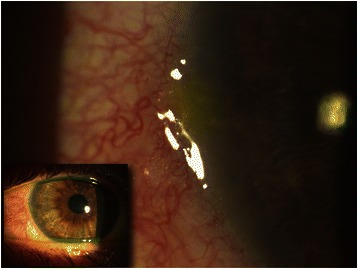
Digital image of the right eye (*small picture*). Limbal congestion with hyperemia and fluorescein staining of an epithelial defect overlying a marginal infiltrate at 9 o’clock limbus (*in magnification*)

Conjunctival congestion with perikeratic hyperemia and light perilimbal conjunctival edema with corneal marginal infiltrates accompanied by epithelial defects were observed in both eyes (Fig. 1). No further ocular alteration was seen. Polymerase chain reaction testing for herpes simplex virus was negative, and cultures of conjunctival secretion were sterile. Complete serum immunology balance, as well screening for autoantibodies, was negative.

**Fig. 2:**
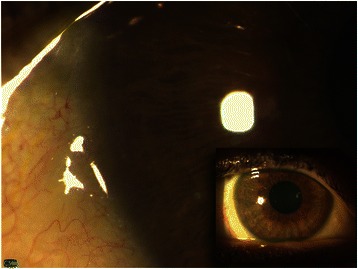
Digital image of the right eye (*small picture*). Evaluation after therapy with topical autologous serum. The corneal epithelium is fully healed and the marginal infiltrate is not detectable

Topical therapy with steroid and antibiotics was started and the right eye was bandaged. Despite initial improvement, the patient’s symptoms worsened after the 13th dose of trastuzumab. As such, we hypothesized a link between HER2 pathway inhibition in the cornea and the ocular side effects. Among the possible strategies to prevent the drug effects on the HER2 pathway in the corneal epithelium, maintenance of sustained activation by administration of a topical serum ligand seemed was proposed as the best solution. Therefore, the 14th administration of trastuzumab was delayed by 1 week, and a sample of peripheral blood was collected. The possibility that a significant concentration of trastuzumab was present in the serum is unlikely due its half-life of 2–12 days. Serum was separated by centrifugation under sterile conditions and stored without dilution at −20 °C. Seven days before the 14th administration of trastuzumab, three drops of serum in each eye was instilled twice per day. Epithelial healing was observed shortly after the onset of treatment with autologous serum, and marginal infiltrates disappeared after 7 days, leaving no haze or scar (Fig. 2). Trastuzumab therapy was restarted without relapse of any of the ocular symptoms. Administration of topical autologous serum was continued in conjunction with trastuzumab for the duration of the patient’s treatment. As a result, no further corneal or ocular disease was observed in post-therapy follow-up.

## Conclusion

Epidermal growth factor receptors are strongly expressed on the surface of basal epithelium cells of the corneal limbus, conjunctive, and throughout the corneal epithelium [12]. EGFR stimulates proliferation and migration of epithelial cells and is generally considered to be the major effector molecule in corneal wound healing [16]. EGFR inhibition has been reported to affect epithelial cell proliferation and stratification during corneal epithelial wound healing, and thus, may play a role in maintaining corneal epithelium thickness [17]. The relative role of the HER2neu member of the EGFR family in maintaining and restoring corneal epithelium integrity is not well known, but is considered crucial in the association of activated-EGFR tyrosine kinase to the migration of corneal epithelial cells [18]. Based on this, we hypothesized that trastuzumab-induced HER2 inhibition may have impaired corneal epithelium turnover in our patient. If correct, then completion for HER2 signaling by providing ligands of HER2 heterodimers could restore normal proliferation and migration of the corneal epithelial cells. Alternatively, if the mechanism of corneal damage had been immunologically mediated by antibody-dependent cell cytotoxicity, receptor internalization following ligand binding would have prevented antigen recognition by the HER2 antibody. Although the patient’s clinical improvement in this case report may also be a result of the seven day delay in further trastuzumab therapy, the lack of corneal damage when trastuzumab therapy was recommenced underlines the protective action on the cornea of the topical autologous serum treatment. Whatever the mechanism of corneal damage, the intervention strategy that we employed allowed us to continue the adjuvant treatment of the patient. It is important to note that this observation does not represent a “proof-of-concept” of pathogenesis of corneal damage during treatment with trastuzumab. In addition, it does not demonstrate a mechanism of local action of patient-derived serum, but the successful clinical result, and the novel approach, are intriguing and warrant in-depth study. Indeed, this treatment method may become more important with time as the HER2 pathway is now a useful target for therapy in 10–20 % of metastatic gastric cancer, whereas for HER+ metastatic breast cancer tratuzumab has been found to be more effective when combined with Pertuzumab, an HER–dimerization inhibitor [19].

As the number of patients undergoing chemotherapy with trastuzumab for HER+ cancer increases it can be expected that the risk of damaging side-effects to corneal tissue will also increase. As such, the successful treatment of limbal lesions and corneal erosion experienced by the breast cancer patient in this case report provide a potentially important new therapy for patients who experience these side effects as a result of trastuzumab therapy.

## Consent

Written informed consent was obtained from the patient for publication of this case report and all accompanying images. A copy of the written consent is available for review by the Editor of this journal.

## Abbreviations

EGFR: epidermal growth factor receptors
HER-2: epidermal growth factor receptors 2
HER-3: epidermal growth factor receptors 3
HER-4: epidermal growth factor receptors 4
